# A THz Receiver with Novel Features and Functionality

**DOI:** 10.3390/s18113793

**Published:** 2018-11-06

**Authors:** Aleksander Sešek, Damjan Berčan, Miha Gradišek, Andrej Švigelj, Janez Trontelj

**Affiliations:** Laboratory for Microelectronics, Faculty of Electrical Engineering, University of Ljubljana, Tržaska 25, Ljubljana, SI-1000 Ljubljana, Slovenia; damjan.bercan@fe.uni-lj.si (D.B.); miha.gradisek@fe.uni-lj.si (M.G.); andrej.svigelj@fe.uni-lj.si (A.Š.); janez.trontelj1@guest.arnes.si (J.T.)

**Keywords:** THz micro-bolometer, micro-bolometer lifetime, sensitivity boosting

## Abstract

The presented THz receiver is based on an antenna coupled titanium micro-bolometer. A new geometrical design improves the robustness and extends the lifetime of the sensor. A study of sensor lifetime using different biasing currents is presented. The lifetime was verified by several tests and over 1000 operating hours. A new micro-bolometer sensitivity measurement algorithm is presented in the paper and measurement results using the proposed algorithm are shown. The new algorithm was developed to be suitable for ATM production testing. In the paper, a novel feature called “sensitivity boosting” is described, together with its influence on sensitivity and lifetime.

## 1. Introduction

Imaging and sensing in different frequency regions is an important requirement in today’s industrial, medicine and security sectors. One of the most researched frequency regions that has attracted a lot of attention in the last years is the terahertz (THz) region. The THz region covers the frequencies from approximately 100 GHz up to 10 THz [1,2]. THz sensors and THz sensor systems have already been in constant development since 1960, but the technology for fabricating sensors capable of operating at room temperature only became mature enough at the beginning of this millennium [3,4]. The sensors are basically divided in two groups: passive sensors [5,6] and active sensors mainly used in Time Domain Spectroscopy (TDS) systems [7]. The sources used for THz illumination [8] still represent the main part of the final THz system price, performance and volume. In the case of solid-state THz sources [9,10,11] they provide a high THz illumination power (from hundreds of mW (10^−3^ W) to several mW) but they also define the THz system central frequency and bandwidth. They can in principle cover only the lower part of the THz frequency region up to 1 THz. On the other hand, TDS sources cover higher portion of THz frequencies from 1 THz to 10 THz, but they offer low power illumination [12] in the µW (10^−6^ W) range. TDS systems use picosecond (10^−12^ s) or femtosecond (10^−15^ s) pulses which provide broadband frequency THz illumination, but as mentioned, with low power levels.

This paper focuses on titanium (Ti) micro-bolometer THz sensors, which output is based on sensing the thermal changes of elemental electrical resistivity. Similar micro-bolometer sensors that are used for imaging in the Infra-Red (IR) region are well known already for decades [13]. IR micro-bolometers receive energy from electro-magnetic (EM) waves that have wavelengths in the range of the bolometer physical dimensions. At the THz frequencies, the wavelengths are longer; therefore, the same principle as in the IR region would not work, as the received “heat” would be immediately annulled by heat transfer into the surrounding space due to the large volume of the micro-bolometer. Monolithic silicon bolometers are used as sensor devices for astrophysical observations [14] where Si micro-bolometers are coated with bismuth and positioned along the E-plane of the waveguide. Such micro-bolometers can reach sensitivities up to 2 × 10^9^ V/W when cooled down to 1 K. Platinum nano-strip bolometers were also used in the W-band as room temperature sensors [15], which are able to achieve high thermal sensitivities of up to 50,000 K/W. As one of the solutions how to receive and measure the incoming radiation power in the THz region, a coupling antenna was proposed in [16]. The sensor operates at room conditions. The proposed method uses a standard Si micromachining process to produce high sensitivity THz sensors.

### Coupled Antenna as Radiation Receiver

**Figure 1:**
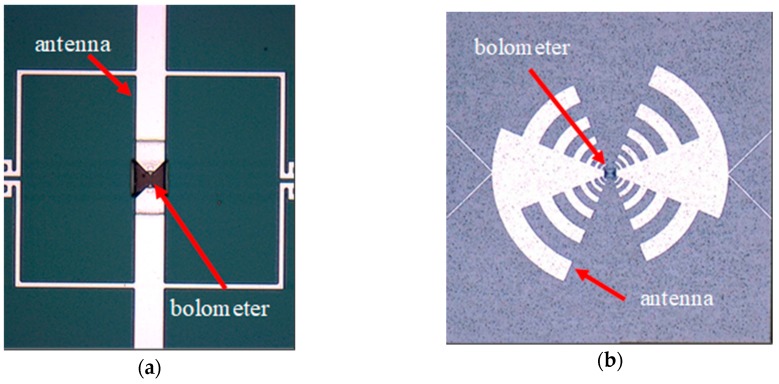
THz sensors for different THz bands: (**a**) A 300 GHz narrow-band antenna with Ti micro-bolometer; (**b**) Wide-band antenna with Ti micro-bolometer (the receiving region is from 80 GHz to 1.1 THz).

A micro-bolometer and coupled antenna were fabricated, using patent pending technology [17]. The resulting sensors are shown in Figure 1a,b.

Figure 1a,b present two versions of several variants, which were designed and fabricated to cover the main THz sub-regions for the different applications [18]. In Figure 1a,b the receiving antenna and micro-bolometer sensing element are marked. The main driving factor of the different antenna band fabrication is in the change of material response (transmittance and absorbance of the materials) [19] and the fact of different absorption of THz waves, due to water vapor in the air. 

**Figure 2:**
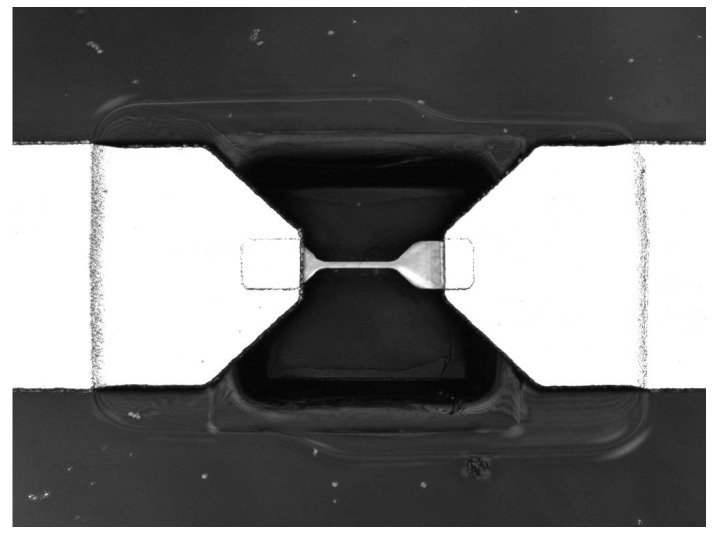
Titanium micro-bridge with cavity.

The received power which is transferred to the Ti micro-bolometer as mentioned before, must be provided carefully, but even so, some power received by the micro-bolometer is lost if the antenna is fabricated on a bulky substrate. To overcome this issue, the antenna is fabricated on a thin 3 µm silicon nitride membrane. The micro-bolometer is then additionally pre-stressed and placed over a small cavity in the membrane to form a bridge. A 3D confocal microscope image of the micro-bridge is presented in Figure 2.

The main power loss is at the antenna connections, which were already designed and their fabrication optimized. To improve the system, two main parameters must be analyzed and measured. Those are sensitivity of micro-bolometer and its lifetime.

## 2. New Method for Micro-Bolometer Sensitivity Measurement

Sensitivity of a micro-bolometer ℜ*_e_* is defined with a simplified equation:

(1) $${ℜ}_{e}\approx \frac{I\cdot TC\cdot R}{G_{th}}$$

where *I* is the biasing current, *TC* is the temperature coefficient of the micro-bolometer material, *R* is a sensor resistance and *G_th_* is the thermal conductivity of the sensor. From Equation (1) it can be seen that for boosting sensor sensitivity, without changing its geometry, only the material (*R*, *TC*, *I* would change) or biasing current (would have influence on *I*) can be changed. As the material properties were investigated precisely [20], the boosting can be done only with increasing the biasing current. The maximal biasing current *I*_0_ can be calculated by the equation:

(2) I0=GthR0·TC

By definition [21] the temperature when *I*_0_ is applied goes to infinity. For typical titanium thermistors with TC 0.2%/K, the biasing current should not exceed 35% of *I*_0_. The margin for our operating bias current *I* was therefore defined as a quarter of the maximal bias *I*_0_. As the increase of *I* to levels higher than *I*_0_/4 has an impact on sensor lifetime, several measurements with different sensors were done to find optimal value of *I* for sensitivity boosting.

The main reason for a new method proposal is the rather complex and unsuitable basic method already used at the end of fabrication line for Automatic Test Measurements (ATM), where improvements of measuring time and precision are needed.

### New Method for Sensitivity Measurement and Calculation

The new method is based on the following basic equation:

(3) $$V_{1}=R_{1}\cdot I_{1}$$

where *V*_1_, *R*_1_ and *I*_1_ are initial values and:

(4) $$V_{2}=R_{2}\cdot I_{2}$$

where *V*_2_, *R*_2_ and *I*_2_ are values after applying a known current step Δ*I* to the bolometer. The equations can be further evolved as:

(5) $$V_{2}=R_{2}\cdot I_{2}=\left(R_{1}+\Delta R\right)\cdot (I_{1}+\Delta I)=R_{1}I_{1}+R_{1}\Delta I+\Delta R(I_{1}+\Delta I)$$

where Δ*R* is the change of micro-bolometer resistance due to heating. The voltage difference on the bolometer through the measurements can be derived from Equations (3) and (5):

(6) $$\Delta V=V_{2}-V_{1}=R_{1}\Delta I+\Delta R(I_{1}+\Delta I)=R_{1}\Delta I+\Delta RI_{2}$$

The only important part for ℜ*_e_* of this voltage difference is:

(7) $$\Delta V=\Delta RI_{2}$$

As the sensitivity is defined as the change of voltage on micro-bolometer divided by received power, equations for the initial power on the micro-bolometer and each step should also be given.

Initial power is described as:

(8) $$P_{1}=V_{1}\cdot I_{1}$$

and power at the following step is stated as:

(9) $$P_{2}=V_{2}\cdot I_{2}=\left(V_{1}+\Delta V\right)\cdot \left(I_{1}+\Delta I\right)=V_{1}I_{1}+\Delta VI_{1}+\Delta I\left(V_{1}+\Delta V\right)$$

The power change on the micro-bolometer can be now derived from Equations (10) and (11) as:

(10) $$\Delta P=P_{2}-P_{1}=\Delta VI_{1}+\Delta I\left(V_{1}+\Delta V\right)=\Delta VI_{1}+\Delta IV_{2}$$

and after including the following term:

(11) $$I_{1}=I_{2}-\Delta I$$

the final power difference can be stated as:

(12) $$\Delta P=\Delta V\left(I_{2}-\Delta I\right)+V_{2}\Delta I=\Delta VI_{2}-\Delta V\Delta I+V_{2}\Delta I$$

Finally, the micro-bolometer sensitivity ℜ*_e_* can be stated as:

(13) $${ℜ}_{e}=\frac{\Delta V}{\Delta P}=\frac{\Delta RI_{2}}{\Delta VI_{2}+V_{2}\Delta I-\Delta I\Delta V}$$

or for each sequential step, knowing that the Δ*I*Δ*U* part can be neglected due to its low value, we can write:

(14) $${ℜ}_{e}=\frac{\Delta V}{\Delta P}=\frac{\Delta RI}{\Delta VI+V\Delta I}$$

Equation (13) includes the contribution of voltage change due to the TC of resistance and the contributions of power changes due to changes of voltage and current. With this method, all micro-bolometers’ sensitivity was calculated and it was proved to be accurate and suitable for using at a wafer test level.

## 3. Results

In this section, the results of micro-bolometer resistance and sensitivity measurements will be presented, using the method proposed in the previous section. Then the lifetime measurements of micro-bolometers, operating on room temperature is presented. The sensitivity boosting method and measurements, using different currents, obtained by lifetime measurements are shown. The section concludes with the new micro-bolometer design proposal, which allows higher biasing currents and has a lower heat dissipation factor.

### 3.1. Measurement of Micro-Bolometer Sensitivity

To prove the method and measure the resistance change and sensitivity of micro-bolometers, the results of four different micro-bolometers, representing an average element from one of the fabricated lots were determined. Table 1 presents the sensor data, obtained at the end of the fabrication line at the final control point. The table includes only data of lots used for sensitivity measurement at room temperature T_a_ = 25 °C.

**Figure 3:**
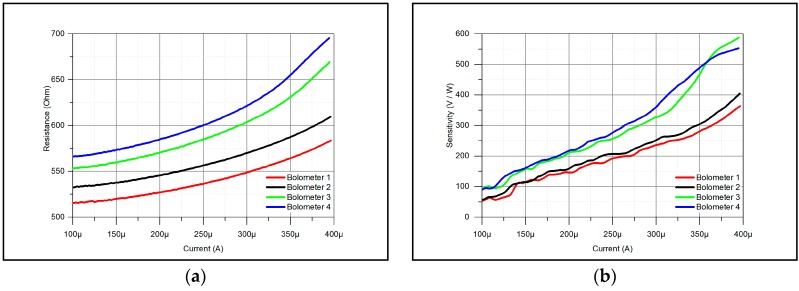
Averaged measurement results: (**a**) resistance; (**b**) sensitivity.

From the table it can be seen that for the measurements, micro-bolometers with different initial resistance and maximal biasing current were chosen. The main reason is to cover different marginal cases. The resistances of the micro-bolometers in the whole batch can reach values, which are not included in this table. Such sensors are not used regularly, but for specific types of applications. All micro-bolometers are made from titanium, with *TC* = 0.13% and they have the same dimensions—12 µm × 1.2 µm and they are 15 nm thick on average. The averaged results are shown in Figure 3.

The first several measured points are not shown due to unstable measurements. From Figure 3a, it can be seen that the resistance curves match the expected curves and the measured values in Table 1. In addition, the sensitivity matches the values from Table 1. From this, it can be concluded that the proposed method is correct and applicable for ATM micro-bolometer sensitivity testing. It can be also concluded from Figure 3b that the sensitivity of the device can be doubled, if the current is raised by a factor of two. This provides a good opportunity for sensitivity boosting—the change of biasing current can double the sensitivity in the requested moment. As the biasing current increase is reflected in the device lifetime and as sensitivity boosting was one of the goals, the measurement of micro-bolometer lifetime was performed. These lifetime measurement results are presented in the next section.

### 3.2. Micro-Bolometer Lifetime Measurements

**Figure 4:**
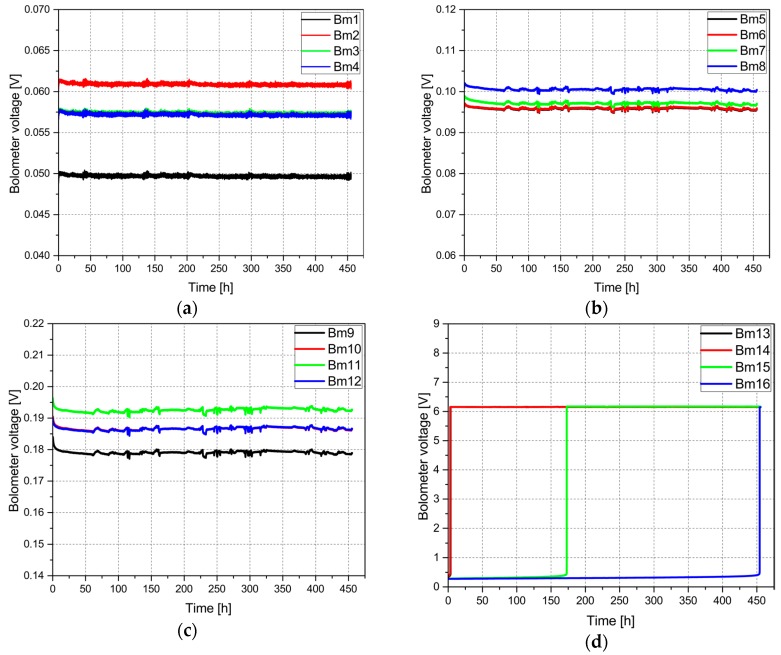
Lifetime measurement results for 16 micro-bolometers. Measured as four micro-bolometers together at: (**a**) 100 µA bias current; (**b**) 200 µA bias current; (**c**) 400 µA bias current and (**d**) 600 µA bias current.

Sixteen micro-bolometers—Bm1 to Bm16—representing the average sensing element as in the previous section, were tested with four different biasing currents to determine the micro-bolometer lifetime. The testing results are shown in Figure 4.

In Figure 4, the results of the micro-bolometers connected to different biasing currents, placed in a controlled temperature chamber, are shown. The cases presented in Figure 4a–c show duplication of micro-bolometer voltage due to duplication of the biasing current for each case. In addition, slight temperature variations can be noticed during the measurements due to the ventilation in the chamber switching ON and OFF.

The last case (Figure 4d) shows how the micro-bolometers were burned out—the Zener protection diode (V_Z_ = 6.2 V) limited the voltage in that case. First, the micro-bolometers Bm13 (black) and Bm14 (red) were burned, then after 170 h Bm15 and at the end of monitoring at 455 h the last one, Bm16, was also destroyed.

All the monitoring of bolometer operation at lower currents were longer (approximately 1000 h), but since the last of the micro-bolometers biased with 600 µA was burned after 455 h, all results in Figure 4 were trimmed accordingly. From the measured results it can be concluded that the “boosting” current of 400 µA can be used without any influence on the micro-bolometers.

### 3.3. Sensitivity Boosting Method

Usually, imaging sensors are assembled in imaging arrays or lines. Sensitivity boosting is normally provided for the whole array or it is obtained using additional optical lenses. In the bottom part of the THz frequency region, where the presented sensors are used, conventional optics for visual light is not applicable. The usage of custom-made THz optics can be applied but it also boosts the signal for the whole imager area. There is no possibility for boosting the sensitivity of a detailed part of an image. As it was already mentioned before, the sensitivity boosting of the presented THz sensors can be done by switching the biasing current of a single element between two values—a basic one for normal screening and a higher value for high sensitivity screening. This can be done for those pixels where a high sensitivity image view is needed, and not necessarily for the whole area. The values of the current should be still in the micro-bolometer safe operation region not to cause sensor burnout. 

**Figure 5:**
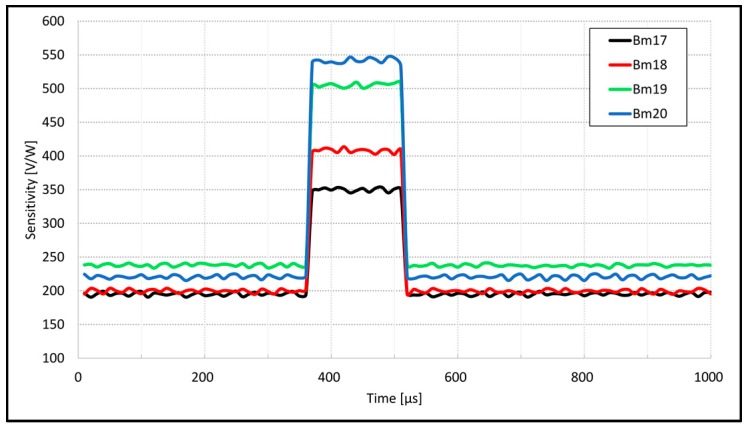
Sensitivity boosting measuring results.

Figure 5 presents the results of sensitivity measurements of four micro-bolometer sensors (Bm17–Bm20) with changed biasing for a certain amount of time. The sensors were taken from different lots. The boosting can be done only for short time period and image should be saved at that moment. Even if the THz sensors survived operation for a long time at higher current, their lifetime would be shorter. The basic biasing current used in this case was 200 µA for normal screening mode measurements and 400 µA for high sensitivity mode. The high sensitivity turn-on time was at 360 µs and turn-off at 510 µs. The rise and fall times for current switching were chosen to be around 1 µs, allowing the sensor to heat up or cool down as the sensor thermal time constant is 0.5 µs [18]. It can be noted that the sensors have different base sensitivity, as well as high sensitivity. This is in the majority of cases due to different thermal constants and initial resistances. As the sample sensors were taken from different lots, this behavior is expected. The values of base sensitivities are around 220 V/W and at higher current value are more scattered—from 350 V/W up to 540 V/W. The ratio between basic current and boosting current and basic sensitivity and boosted sensitivity is not the same for all sensors, mainly due to their different thermal constants and because of small variations in micro-bolometer dimensions. The proposed sensitivity measuring method will give enough data to predict and react accordingly to equalize the sensitivity and prevent possible burnouts. All sensors were measured at room temperature without vacuum encapsulation. In the case of encapsulation in vacuum, the sensitivities would be at least doubled.

One of the main issues when using higher current values is possible sensor damage, which can happen at high current switching. From the previous THz sensor thermal behavior research [22] it can be derived that the micro-bolometer has a main heating point in the middle of the micro-bridge, as that is where the highest current density and the lowest thermal conductance are. Also in all cases, when the bolometer was burned out, this happened in that point. That was the reason to study a new micro-bolometer design, which would decrease the thermal stress on the micro-bridge and therefore distribute the heating across the whole area of the micro-stripes without losing any sensitivity. The new design description is given in the following section.

### 3.4. New Micro-Bolometer Design

The reasons of the new design were already highlighted in the previous section. The main purpose of the new design was to lower the current density in the middle of the micro-bridge without losing the sensitivity.

**Figure 6:**
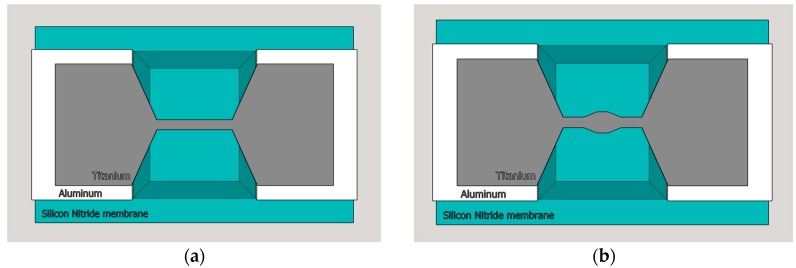
New titanium micro-bridge design with bridge expansion and cavity (**a**) current Ti micro-bolometer design (**b**) proposed new design.

From the fabrication point of view, there are not a lot of possibilities for expanding the central area of the micro-bridge. As the micro-bolometers are fabricated on a 2–3 µm silicon-nitride membrane that is additionally etched below the bridge, expansion below the flattened central plane is not achievable with simple fabrication steps. The thickening above the flattened middle structure of the current design, which can be seen on Figure 6a, would require an additional mask and at least three additional fabrication steps. Therefore, the only straightforward solution was to widen the central area by approximately 20% to compensate for the current density and thermal conductance differences. The proposed design can be seen in Figure 6b. The proposed new THz sensor is currently in fabrication and has not been measured yet.

**Figure 7:**
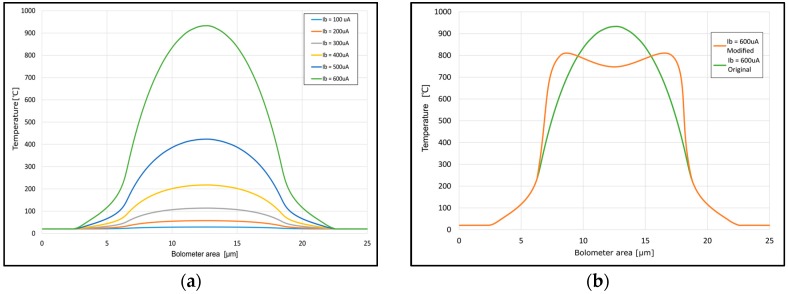
Simulation results of temperature distribution: (**a**) temperature distribution in the current micro-bolometer design at different biasing currents, (**b**) temperature distribution of current and proposed micro-bolometer design at 600 µA biasing current.

To prove the proposed design, simulations were done in Comsol Multiphysics^®^ (Comsol Inc., Burlington, MA, USA). The micro bolometer structure was designed in Solidworks^®^ (Dassault Systemes SolidWorks Corporation, Waltham, MA, USA), based on the GDS layout file. The basic design presented in Figure 6a was simulated first, using several biasing currents as shown in Figure 7a. The temperature in the central area, when all thermal flows are included, can reach up to 900 °C at 600 µA biasing current.

From the Figure 7b, it can be seen that when the design shown in Figure 6b with wider micro-bolometer central area is used, the temperature decreases due to the lower current density. It can be concluded that in this case, a higher biasing current could be used and therefore higher sensitivity can be reached.

## 4. Discussion and Conclusions

The proposed sensitivity measuring method turned out to be fast and precise. The micro-bolometers have stable behavior and do not vary within a single lot. Further investigation is needed to determine how to stabilize the behavior between the LOTs, but the sensitivity measurement method will remain the same.

The average sensitivity of the THz micro-bolometer sensors at 200 µA is typically 200 V/W and can be increased by a biasing current increase. However, the maximal level of biasing current tends to be approximately 500 µA. The average resistance is 550 Ω. The future research will be focused on the method that will ensure constant micro-bolometer resistance to maintain a good match with the antenna impedance at the selected frequency. This is also important when changing the micro-bolometer dimensions. Future work will be therefore oriented toward future modifications of our micro-bolometer design to reach the best sensor sensitivity and longer lifetime, thus enabling sensitivity boosting.

## Figures and Tables

**Table 1:** Micro-bolometer parameters.

Marking	Lot No.	*R*_0_^1^ [Ω]	*I*_0_ [µA]	*G_th_* [µW/K]	ℜ*_e_* [V/W]
Bolometer 1	A02	511	1038	0.69	264
Bolometer 2	A06	533	1066	0.76	257
Bolometer 3	A07	553	971	0.66	282
Bolometer 4	A09	567	875	0.54	313

^1^ The value is measured at 100 µA bias current.
